# XUV-Initiated Dissociation Dynamics of Molecular Oxygen (O_2_)

**DOI:** 10.1021/acs.jpca.1c06033

**Published:** 2021-11-17

**Authors:** Marc Rebholz, Thomas Ding, Lennart Aufleger, Maximilian Hartmann, Kristina Meyer, Veit Stooß, Alexander Magunia, David Wachs, Paul Birk, Yonghao Mi, Gergana Dimitrova Borisova, Carina da Costa Castanheira, Patrick Rupprecht, Maia Magrakvelidze, Uwe Thumm, Sebastian Roling, Marco Butz, Helmut Zacharias, Stefan Düsterer, Rolf Treusch, Günter Brenner, Christian Ott, Thomas Pfeifer

**Affiliations:** † Max-Planck-Institut für Kernphysik, Saupfercheckweg 1, Heidelberg 69117, Germany; ‡ Cabrini University, 610 King Of Prussia Road, Radnor, Pennsylvania 19087, United States; § Kansas State University, 212 Cardwell Hall, Manhattan, Kansas 66506, United States; ∥ Physikalisches Institut, Westfälische Wilhelms-Universität, Wilhelm-Klemm-Str. 10, Münster 48149, Germany; ⊥ Deutsches Elektronen-Synchrotron, Notkestraße 85, Hamburg 22607, Germany

## Abstract

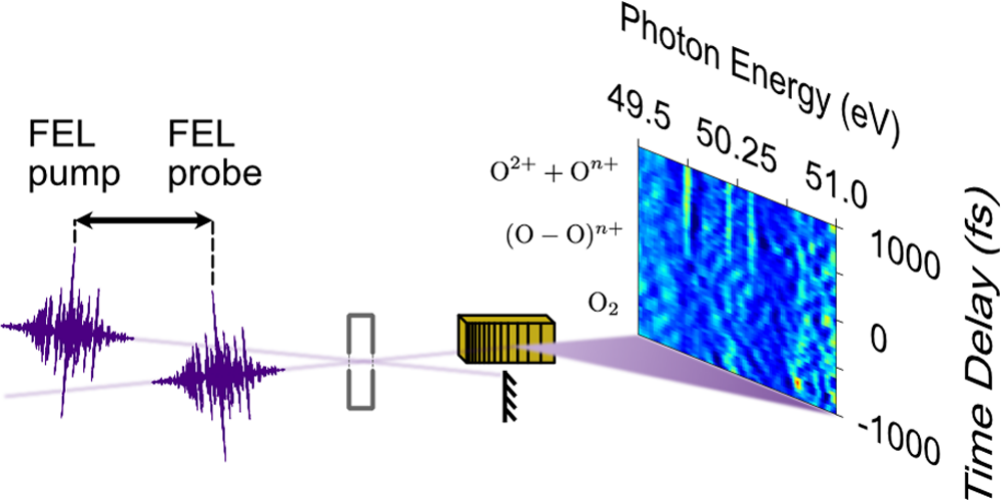

We performed a time-resolved spectroscopy experiment on the dissociation of oxygen molecules after the interaction with intense extreme-ultraviolet (XUV) light from the free-electron laser in Hamburg at Deutsches Elektronen-Synchrotron. Using an XUV-pump/XUV-probe transient-absorption geometry with a split-and-delay unit, we observe the onset of electronic transitions in the O^2+^ cation near 50 eV photon energy, marking the end of the progression from a molecule to two isolated atoms. We observe two different time scales of 290 ± 53 and 180 ± 76 fs for the emergence of different ionic transitions, indicating different dissociation pathways taken by the departing oxygen atoms. With regard to the emerging opportunities of tuning the central frequencies of pump and probe pulses and of increasing the probe–pulse bandwidth, future pump–probe transient-absorption experiments are expected to provide a detailed view of the coupled nuclear and electronic dynamics during molecular dissociation.

## Introduction

Oxygen plays a central role in the metabolism of many life forms on earth^1^ as well as in combustion processes.^2^ Furthermore, it shields the earth from ultraviolet radiation in the form of the ozone layer.^3^

In addition to its fundamental importance, as a diatomic molecule, oxygen can serve as a model system for the experimental and theoretical study of nuclear wave-packet dynamics.^4−7^ In recent years, such experiments have mostly been conducted using few-cycle near-infrared (NIR) laser pulses in time-resolved pump–probe spectroscopy studies.^6−9^ However, the intense electric field of the NIR pulses introduces strong couplings between individual electronic states. Therefore, instead of the fundamental field-free potential energy curves (PECs), the field-dressed PECs are probed.^4^

The introduction of extreme ultraviolet (XUV) free-electron lasers (FELs)^10^ as intense sources of XUV radiation made XUV-pump–XUV-probe schemes possible. Hereby, the first FEL pulse initiates a nuclear wave packet by ionizing the target molecule, while the second FEL pulse probes the evolution of the system. Because of the high photon energy of the XUV radiation, a single photon is sufficient for the ionization, which is in contrast to a multiphoton excitation/ionization with NIR radiation. In this way, the evolution of the nuclear wave packet on the unperturbed potential energy curves can be probed by the second XUV pulse. This can be done by either recording photoelectron^11^ or fragment kinetic energy release^12,13^ spectra of the target but also via the detection of the molecule’s absorption spectrum.^14,15^

Photoelectron/photofragment ion spectroscopy gives detailed information on all produced fragments and electrons including their kinetic energies over a broad bandwidth.^16−18^ It is even possible to reconstruct the three-dimensional (3D) momentum distribution of produced fragments or ejected photoelectrons. However, it is only possible to detect the final states of a fragment, which could have decayed on its way from its origin to the detector. In contrast, transient absorption spectroscopy (TAS) allows for the continuous probing of a dissociating molecule’s electronic structure and its resonant transitions, right at the time when the probe pulse arrives, within the window of the probe pulse’s spectral width.^19−21^

In this work, we demonstrate the time-resolved spectroscopic access to the electronic structure during the pump-induced interaction through XUV-pump—XUV-probe transient absorption spectroscopy.

## Setup

**Figure 1:**
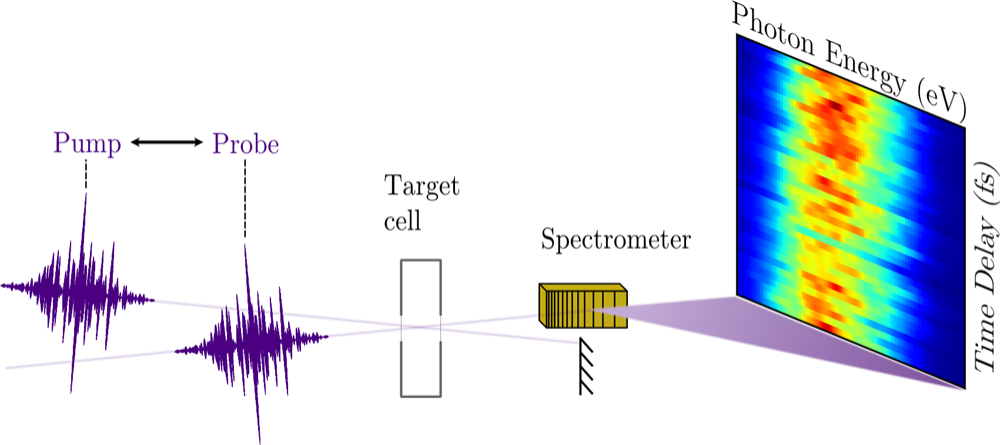
The incident FEL beam is split into two parts via the FLASH BL2 SDU.^22^ The hereby generated pump and probe pulses are focused into a gas cell containing the oxygen target. The spectra of the probe pulses are detected as a function of the pump–probe time delay with a spectral resolution of 35 meV at 50 eV photon energy.

The employed FEL pulses from the free-electron laser in Hamburg (FLASH)^10^ have a photon energy of 50 eV with a bandwidth of ∼0.5 eV (full width at half-maximum (fwhm)), a pulse duration of 65 ± 15 fs, and an intensity in the 10^13^ W cm^–2^ regime. Pump and probe pulses are generated by geometrically splitting the incoming FEL pulses in the split-and-delay unit (SDU) at beamline BL2 at FLASH.^22^ The spectra of the probe pulses, transmitted through a gas cell that contains a moderately dense sample of molecular oxygen (backing pressure: 41 mbar; target length: 4 mm; focus size: 25 μm), are detected as a function of the pump–probe time delay with a step size of 40 fs (see Figure 1). The instrument response function of the setup is determined to be 65 ± 15 fs fwhm via an independent measurement in a neon gas target under the same experimental conditions.^23^ This measurement also allowed us to determine the temporal overlap between pump and probe pulses with an accuracy better than 10 fs.^23^ A more detailed description of the experimental setup can be found in ref (14).

## Results

**Figure 2:**
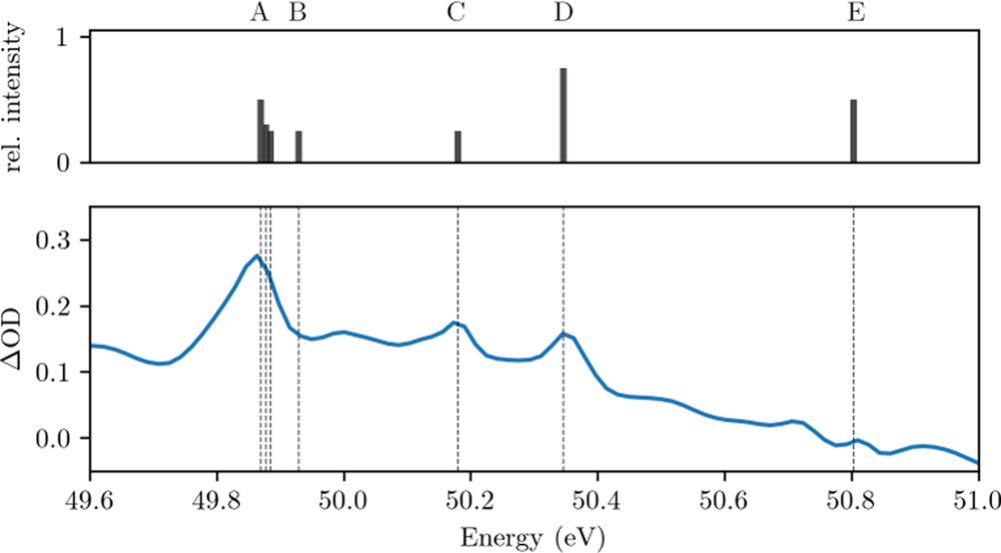
ΔOD showing the pump-induced absorption changes. It is calculated according to eq 1. Letters A through E refer to the O^2+^ transitions listed in Table 1. Figure adapted from ref (25).

The measured quantity in the presented transient absorption study is the spectrum of the FEL probe pulse transmitted through an oxygen gas sample. In the presence of the FEL pump pulse, the absorption signal is modified through the interaction with the highly intense XUV light. In Figure 2, the differential optical density (ΔOD) is depicted. It shows the changes the pump pulse induces within the target sample and is calculated according to
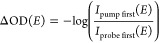
1where *I*_pump first_(*E*) is the spectrum of the probe pulse when the pump pulse precedes it by more than 400 fs. In this case, the pump has modified the response of the system, which is reflected in a change in the absorption of the probe pulse. In the opposite case (*I*_probe first_(*E*)), the probe spectrum is detected as well; however, the pump pulse trails the probe by more than 400 fs. Thus, the detected probe spectrum experiences the unaltered absorption of the oxygen target. The pump-induced absorption changes are calculated according to eq 1 and are shown in Figure 2 (lower panel). Sharp absorption features can be observed in the ΔOD. Additionally, the energetic positions of several transitions of the O^2+^ cation are plotted in the top panel of Figure 2. The spectroscopic assignment is carried out along literature values in ref (24) and is summarized in Table 1. The letters A through E are assigned to individual absorption features for easier identification.

**Table 1:** Parameters of O^2+^ Absorption Lines^24^

transition	term scheme	energy (eV)
A: 2p^2^–2p5d	(g^3^P_2_)–(^3^D_3_^o^)	49.87
A: 2p^2^–2p5d	(g^3^P_1_)–(^3^D_2_^o^)	49.88
A: 2p^2^–2p5d	(g^3^P_0_)–(^3^D_1_^o^)	49.89
B: 2p^2^–2p7d	(^1^D_2_)–(^1^F_3_^o^)	49.93
C: 2s^2^2p^2^–2s2p^2^3p	(^1^D_2_)–(^1^F_3_^o^)	50.18
D: 2s^2^2p^2^–2s2p^2^3p	(^1^D_2_)–(^1^D_2_^o^)	50.35
E: 2s^2^2p^2^–2s2p^2^3p	(^1^D_2_)–(^1^P_1_^o^)	50.80

**Figure 3:**
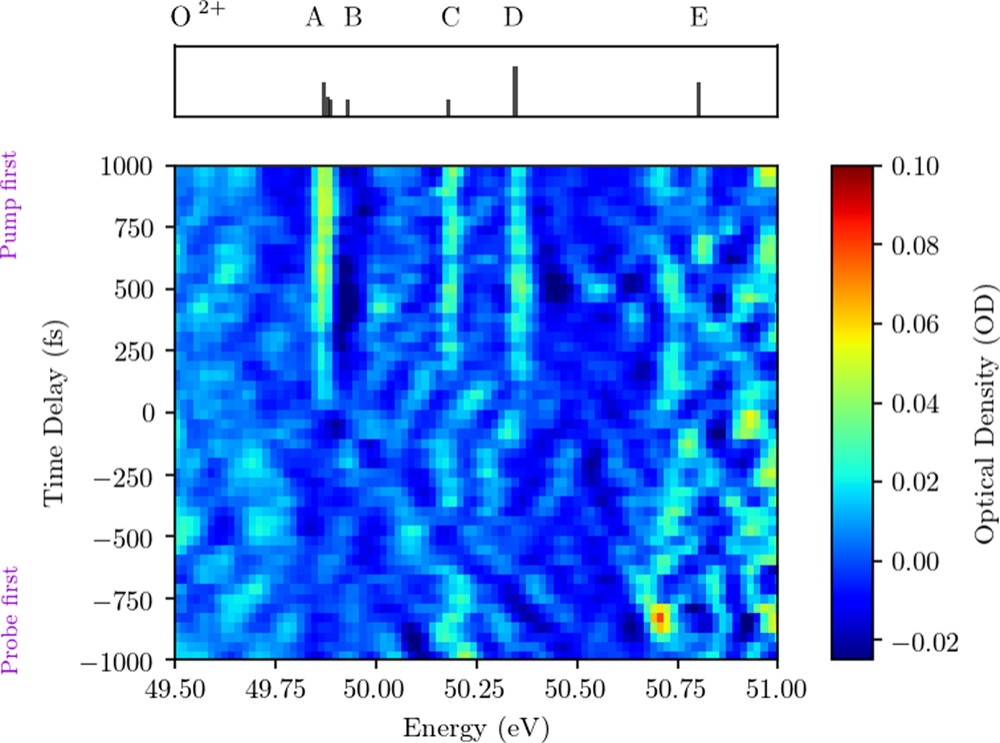
Experimentally measured time-dependent absorption spectra, obtained through the generated Fourier reference spectra. The emergence of several O^2+^ absorption lines, labeled here with A, C, and D (cf. Table 1), is clearly visible over the course of the time delay. Transitions B and E are hidden in the noise floor of the measurement. Note, most prominently visible at negative time delays and in the overlap region, spectrotemporal structures appear, which are an artifact of the Fourier bandpass filter and lead to systematic deviations. Figure adapted from ref (25).

In Figure 3 the time-dependent OD of the oxygen target is shown. In the absence of a suitable measured reference spectrum, a reconstructed reference spectrum *I*_Fourier ref_(*E*, Δ*t*) of the experimentally measured probe transmission spectrum *I*_probe_(*E*, Δ*t*) is calculated for every time-delay step Δ*t* via a Fourier bandpass filter. Therefore, the spectra are Fourier-transformed along their energy axis, and a Gaussian low-pass filter is used to suppress the high-frequency Fourier components stemming from the sharp absorption resonances. These filtered Fourier spectra are transformed back into the spectral domain. In order to mitigate artificial high-frequency components from a Fourier transformation of detector noise outside of the bandwidth of the spectra, the probe spectra are tapered by a cos^2^ function. The employed Gaussian low-pass filter is defined as
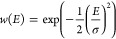
2

Its width σ is chosen to be large enough to still recover the spectral variations of the FEL spectrum but not too large, as otherwise the sharp spectral absorption features in the OD would be removed as well (cf. Figure 3). To avoid systematical effects, below (Table 2) we also performed a comparison for several choices of σ. With the hereby generated Fourier reference *I*_Fourier ref_(*E*, Δ*t*) the OD can be calculated.
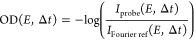
3

**Table 2:** Results of Fitting Eq 4 to the Lineouts A and D

	filter 1	filter 2	filter 3	average
	(σ_1_ = 19 fs)	(σ_2_ = 23 fs)	(σ_3_ = 27 fs)	
τ_A_	300 ± 60 fs	290 ± 50 fs	270 ± 50 fs	290 ± 53 fs
τ_D_	178 ± 79 fs	177 ± 77 fs	186 ± 73 fs	180 ± 76 fs

The emergence of sharp absorption features is clearly visible over the course of the pump–probe time delay (see Figure 3). The upper panel of Figure 3 shows the same O^2+^ resonances as the top panel of Figure 2. Regarding the time-dependent measurement, three distinct absorption features emerge for positive time delays (see Figure 3). The most prominent feature can be assigned to the triplet transition A centered at 49.88 eV, where the 10 meV-scale triplet structure is unresolved at our spectrometer resolution. Additionally, two signatures of the 2s^2^2p^2^–2s2p^2^3p transition at 50.18 eV (^1^D–^1^F) and 50.35 eV (^1^D–^1^D) can be observed, while the remaining absorption lines remain hidden in the noise floor of the data.

**Figure 4:**
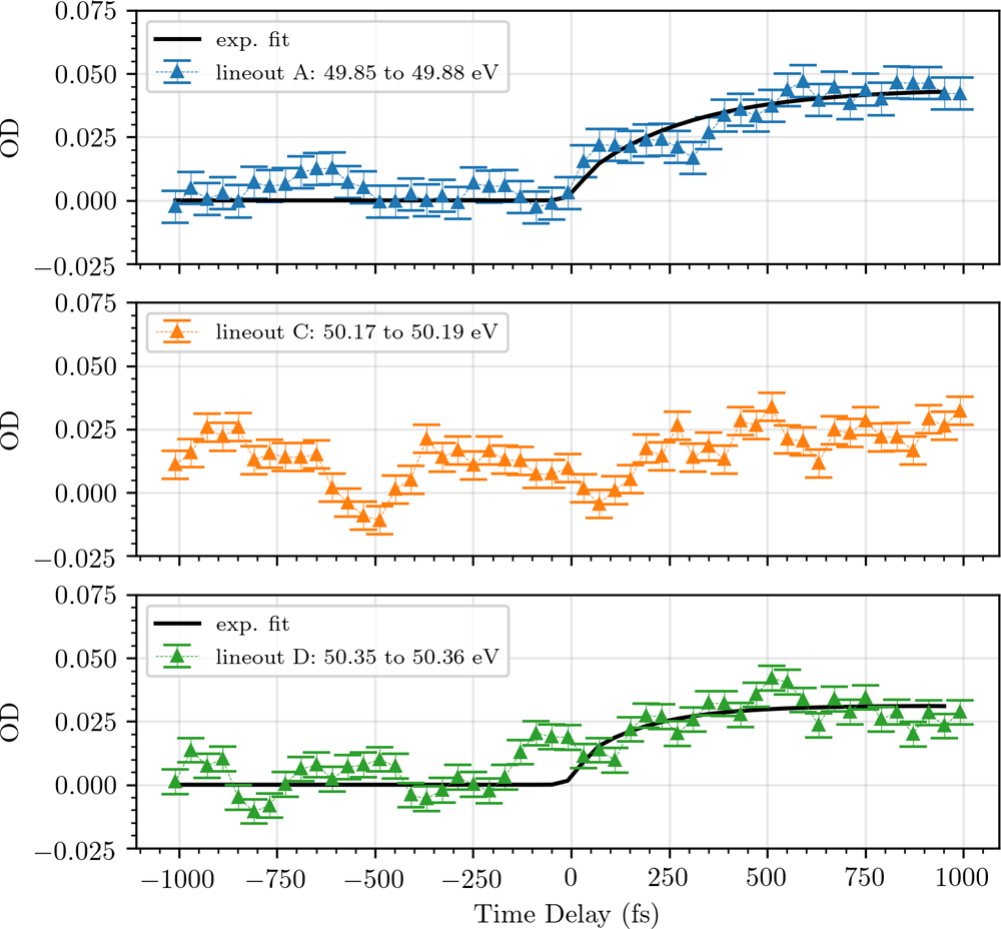
Lineouts of the OD, averaged over the absorption resonances A, C, and D. The lineouts of A and D are fitted with the exponential model introduced in Eq 4, while the statistics of the data does not allow for a sensible fit of lineout C. The results of this fit are summarized in Table 2.

The lineouts through the energy regions of the absorption features A, C, and D can be found in Figure 4. The error bars of the lineouts are calculated by taking additional lineouts through the data in regions with no measured absorption right next to transition A (from 49.74 to 49.81 eV), C (from 50.10 to 50.14 eV), and transition D (from 50.40 to 50.47 eV). The standard deviation of these lineouts is used as a measure for the statistical fluctuation of our experimental data. Lineouts A and D are then fitted with an exponential model to get a measure for the time scales of their emergence. The statistics of the data for lineout C is not sufficient to perform the same fit.
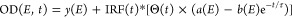
4Here, *y*(*E*) is a time-independent offset, IRF(*t*) is the instrument response function, and Θ(*t*) is the Heaviside step function. The symbol (*) denotes the convolution between instrument response function and the function that describes the dynamics, while (×) denotes multiplication. *a*(*E*) and *b*(*E*) are fit amplitudes, while τ is the time constant of the exponential rise of the absorption line. The fits are performed for three different widths σ of the Fourier filter to get an estimate of its influence on the time scales. The results of the fit are summarized in Table 2. All three results per lineout agree well within the error margins. A strong influence of the width of the Gaussian low-pass filter on the resulting time scales can thus be ruled out.

The emergence of the absorption resonance A of the 2p^2^–2p5d triplet with a time constant of τ̅_A_ = 290 ± 53 fs is reproduced reasonably well by the exponential fit model [Eq. 4]. Absorption resonance D (2s^2^p^2^–2s2p^2^3p) arises with a time constant of τ̅_D_ = 180 ± 76 fs. As can be seen in Figure 4, there is an increase in resonance line D already 100 fs before the temporal overlap of pump and probe pulses. This is most likely linked to an artifact of the Fourier filter (cf. Figure 3). The fit model mostly ignores this artifact through the Heaviside step function Θ(*t*). Excluding the artifact from the fit (time-delay region between −170 and +30 fs) returns τ_D_^*^ = 150 ± 45 fs, which agrees with the original value within error bars.

## Discussion

As was discussed above, we observe several absorption lines of the O^2+^ cation stemming from different ground-state configurations (cf. Table 1). In addition we can time-resolve the emergence of these lines (see Figure 4).

**Figure 5:**
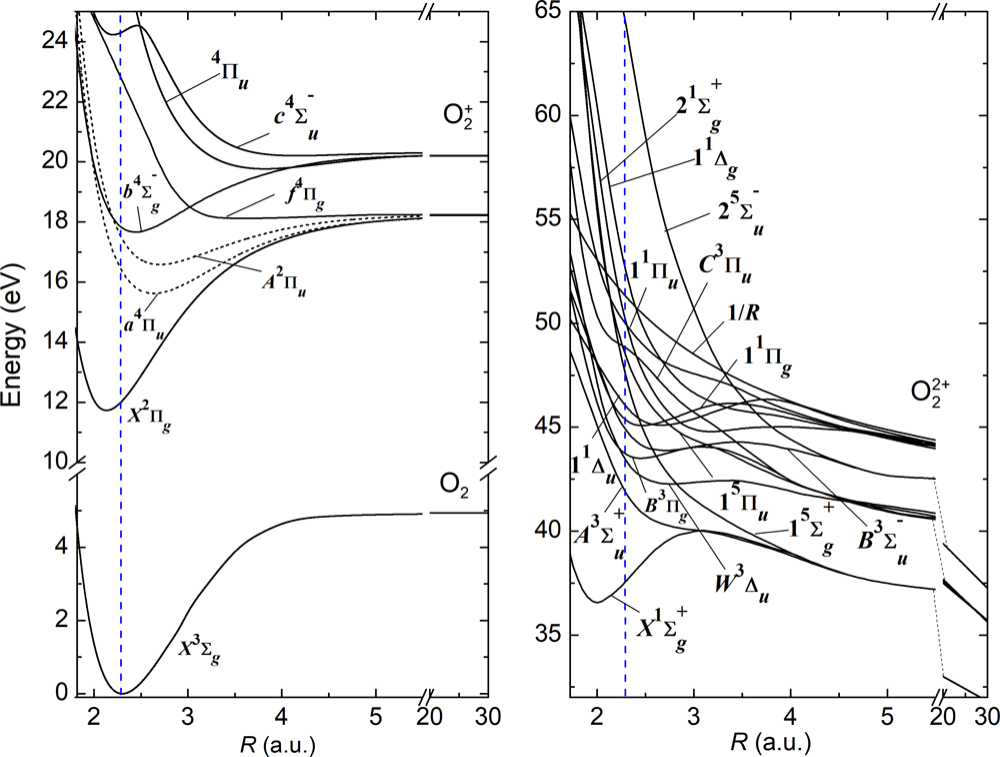
Oxygen PECs, reprinted from ref (13). The blue dashed line marks the equilibrium distance of neutral oxygen. The mainly populated PECs as identified in ref (13) are drawn as dashed curves.

The high intensity of the pump pulse leads to the production of singly and doubly charged molecular cations^26^ (O_2_^+^ and O_2_^2+^). Hereby, multiple bound and dissociative molecular states are populated (see Figure 5), including states with a predissociative character.^27^ Their relevance as intermediate states for measured kinetic energy release (KER) distributions of charged fragments in an XUV pump–probe experiment has been previously discussed.^13^

Using transient absorption spectroscopy we are able to directly follow in the experiment the molecular dissociation dynamics by tracking the appearance times for electronic transitions in a particular molecular fragment. This holds promise for molecular TAS to reveal the entire molecular dissociation dynamics, revealing the evolving electronic structure from the bound molecule to the separated fragment limit for specific fragmentation channels. In previous measurements of KER distributions, this information could be extracted indirectly only and depends strongly on theoretical modeling.

Nuclear motion along these various PECs may be initated, at least in principle, through the pump-pulse trigger. In the following, we classically compute the internuclear motion along all potential energy curves that lead to neutral O and charged O^+^ atomic fragments. Hereby it should be noted that the probe pulse may further ionize these fragments, generating O^2+^, which finally leads to the detection of the observed spectroscopic lines.

To get an estimate for the time scales of the different dissociation pathways, a simple model is employed. A classical particle with the reduced mass μ of the oxygen molecule and an initial velocity *v*_0_ = 0 is started on every PEC from Figure 5 at the equilibrium distance of the oxygen molecule of *r*_0_ = 2.28 au. The evolution of this particle on the individual PECs is calculated by a numerical integration along the internuclear distance *r*. Therefore, the reaction coordinate *r* is discretized into equally sized incremental steps Δ*R* = *r*_*i*_ – *r*_*i*–1_. From the potential energy curves *V*(*r*), the acceleration *a*_*i*_ of the particle can be calculated at every point *i* along the reaction coordinate . From this, the time
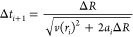
5that is needed to go from one point *i* of the reaction coordinate to the next (*i* + 1) is calculated.

The velocity *v*(*r*_*i*_) of the particle at the beginning of every step is calculated from the kinetic energy
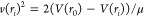
6where *V*(*r*_0_) is the particle’s initial potential energy, and *V*(*r*_*i*_) is its potential energy at every step *i*. To calculate the total elapsed time that is needed to arrive at a certain internuclear distance, the incremental time steps Δ*t*_*i*_ are summed up.

**Figure 6:**
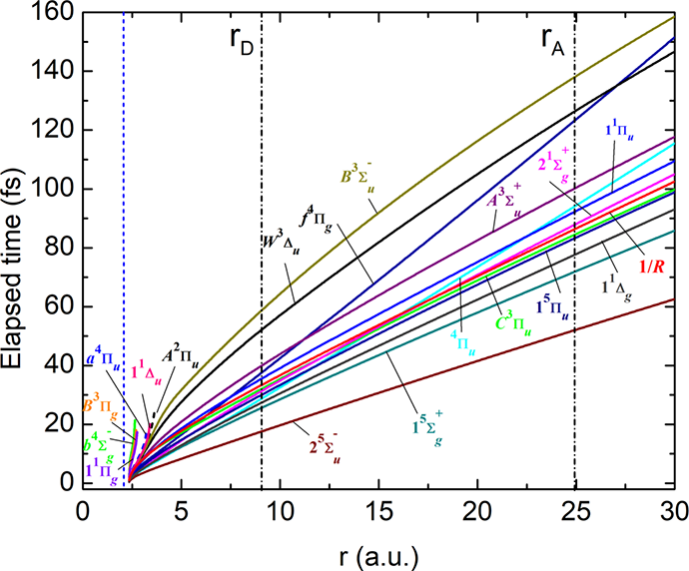
Total travel time of a particle with the reduced mass of oxygen initially placed at the equilibrium internuclear distance of the neutral molecule at *r*_0_ = 2.28 au (blue dashed vertical line) for each PEC. The size of the Rydberg orbitals of the two excited states of transitions A and D are marked as black dashed-dotted vertical lines. For the cases the particle is trapped in a bound PEC [cf. Figure 5], the calculation of the travel time is aborted at the first turning point (e.g., as marked for A^2^Π_u_).

In Figure 6, we plot the total travel time as a function of the internuclear distance for each individual potential energy curve. To estimate the internuclear distance at which we expect the observed atomic transitions, we calculate in hydrogenic approximation the Bohr radii *r* = *n*^2^*a*_0_ of the two excited states involved in the transitions A (*n* = 5) and D (*n* = 3), *r*_A_ and *r*_D_, respectively (marked as black dashed-dotted vertical lines in Figure 6).

Direct dissociation along these isolated curves thus seems not to support the experimental finding in Figures 3 and 4, where individual atomic lines are exponentially rising only within a time scale of a few 100 fs. A different mechanism may thus be at play, for example, predissociation as discussed in refs (27−30). Indeed, in Figure 5, we also observe the trapping of the classical particle in a potential well for several PECs of the O_2_^+^ molecular cation, corresponding to long-lived bound states. Some of these states are known to predissociate.^27−30^ A mechanism that involves the coupling of different molecular PECs is obviously not captured in this simplified model calculation and requires further investigation. Interestingly, the experimental finding of two different appearance times suggests two different delayed or predissociation channels that lead to the formation of the corresponding transitions.

## Conclusion

By using femtosecond XUV-pump–XUV-probe transient absorption spectroscopy, we are able to time-resolve and spectroscopically identify the appearance of individual atomic transitions in O^2+^, after triggering the dissociation of O_2_ by a 50 eV FEL pump pulse. With a spectral resolution of ∼35 meV at 50 eV we have identified several transitions in O^2+^ with different initial-state electronic configurations, which exponentially rise with different time scales. These time scales are of the order of a few 100 fs. They are substantially longer than those expected from a simple motion along individual PECs, which has been found through a comparison with a classical model. A possible explanation for this delayed rise could be the contribution of predissociative states.

This first experiment on XUV-pump–XUV-probe transient absorption spectroscopy of oxygen molecules still has limitations, mainly in temporal resolution and bandwidth. Nevertheless, we were able to measure the onset of different atomic transitions after the dissociation of the molecule as a function of the pump–probe time delay. We demonstrate the time-resolved spectroscopic access to the electronic structure of the molecule during the pump-induced interaction through XUV-pump—XUV-probe transient absorption spectroscopy. The extension of this technique to a broader probe spectrum and shorter probe pulses, possibly in technologically very challenging combination with a high-harmonic probe following the XUV FEL pump, will substantially increase the possibilities of this experimental scheme, since spectroscopically selective access to a much wider range of intermediate and final states will be achievable in this way.
